# Methyl probes in proteins for determining ligand binding mode in weak protein*–*ligand complexes

**DOI:** 10.1038/s41598-022-13561-y

**Published:** 2022-07-04

**Authors:** Biswaranjan Mohanty, Julien Orts, Geqing Wang, Stefan Nebl, Wesam S. Alwan, Bradley C. Doak, Martin L. Williams, Begoña Heras, Mehdi Mobli, Martin J. Scanlon

**Affiliations:** 1 Medicinal Chemistry, Monash Institute of Pharmaceutical Sciences, Monash University, 381 Royal Parade, Parkville, VIC 3052 Australia; 2 ARC Centre for Fragment-Based Design, Monash Institute of Pharmaceutical Sciences, Monash University, 381 Royal Parade, Parkville, VIC 3052 Australia; 3 Sydney Analytical Core Research Facility, The University of Sydney, Sydney, NSW 2006 Australia; 4 Department of Pharmaceutical Sciences, University of Vienna, Althanstrasse 14, 1090 Vienna, Austria; 5 La Trobe Institute for Molecular Science, La Trobe University, Melbourne, VIC 3085 Australia; 6 Centre for Advanced Imaging, The University of Queensland, St Lucia, QLD 4072 Australia

**Keywords:** Solution-state NMR, X-ray crystallography, Drug discovery

## Abstract

Structures of protein–ligand complexes provide critical information for drug design. Most protein–ligand complex structures are determined using X-ray crystallography, but where crystallography is not able to generate a structure for a complex, NMR is often the best alternative. However, the available tools to enable rapid and robust structure determination of protein–ligand complexes by NMR are currently limited. This leads to situations where projects are either discontinued or pursued without structural data, rendering the task more difficult. We previously reported the NMR Molecular Replacement (*N*MR^2^) approach that allows the structure of a protein–ligand complex to be determined without requiring the cumbersome task of protein resonance assignment. Herein, we describe the *N*MR^2^ approach to determine the binding pose of a small molecule in a weak protein–ligand complex by collecting sparse protein methyl-to-ligand NOEs from a selectively labeled protein sample and an unlabeled ligand. In the selective labeling scheme all methyl containing residues of the protein are protonated in an otherwise deuterated background. This allows measurement of intermolecular NOEs with greater sensitivity using standard NOESY pulse sequences instead of isotope-filtered NMR experiments. This labelling approach is well suited to the *N*MR^2^ approach and extends its utility to include larger protein–ligand complexes.

## Introduction

Structure-based drug design has proven to be very efficient in the hit to lead optimization process^1^. X-ray crystallography is the preferred technique to generate structural data^2^. While crystallization generally works well for high affinity ligands, those that bind weakly can prove more challenging. When the complex does not crystallize, NMR can provide an alternative approach to generate structural data. However, the time required to generate a structure by NMR is often too long for medicinal chemistry projects. In recent years, numerous NMR methods have been developed that report on the binding mode of the ligand^3^. Chemical shift perturbation (CSP)^4–7^, saturation transfer^8^, partial NMR resonance assignment^9^, and pseudocontact shift approaches^10,11^ are a few methods addressing this issue^12–14^. We previously reported a method to derive the structure of a protein*–*ligand binding site called NMR Molecular Replacement, *N*MR^2^. This method can generate accurate structures of a protein*–*ligand binding site within a few days^15–17^. Neither backbone nor sidechain assignments are required for *N*MR^2^. Instead, this method uses semi-ambiguous protein*–*ligand inter-molecular NOEs and intra-ligand NOEs as experimental inputs in conjunction with a starting model of the protein to calculate the bound structure. The structure calculations are based on standard NMR protocols, and the outputs are ranked according to the agreement between the experimental input and the calculated structure. *N*MR^2^ is a systematic method and consequently does not contain any stochastic component. It also does not rely on docking recipes but on a series of robust structure calculation protocols. *N*MR^2^ can be used to solve several different types of complex structures involving strong and weak binders, small molecules, peptides and peptidomimetics^15–17^. The method is also capable of detecting cryptic binding pockets^15–17^. Herein we detail the use of *N*MR^2^ in conjunction with specific methyl labelling schemes in a deuterated background (see Supplementary Fig. S1). The advantage of specific methyl labelling schemes in protein NMR, where the methyl groups are protonated in an otherwise deuterated background, allow much higher molecular weight systems to be studied due to the favourable relaxation properties of methyl groups^18,19^. Additionally, stereospecific labelling of methyl groups is possible, which reduces the ambiguity of protein resonance assignments.

Here, we exemplify the potential of combining the *N*MR^2^ method with a selective methyl labelling technique on a challenging system comprising phenylthiazole **1**, a flexible low-affinity ligand binding to a shallow and dynamic site on the protein. The model protein used in this study is a 21 kDa DsbA enzyme from *Escherichia coli* (*Ec*DsbA) that catalyzes the formation of disulfide bonds in secreted proteins within the periplasm. *Ec*DsbA is essential for bacterial virulence and therefore represents an attractive target for the development of new antibacterials^20^.

## Results and discussion

**Figure 1:**
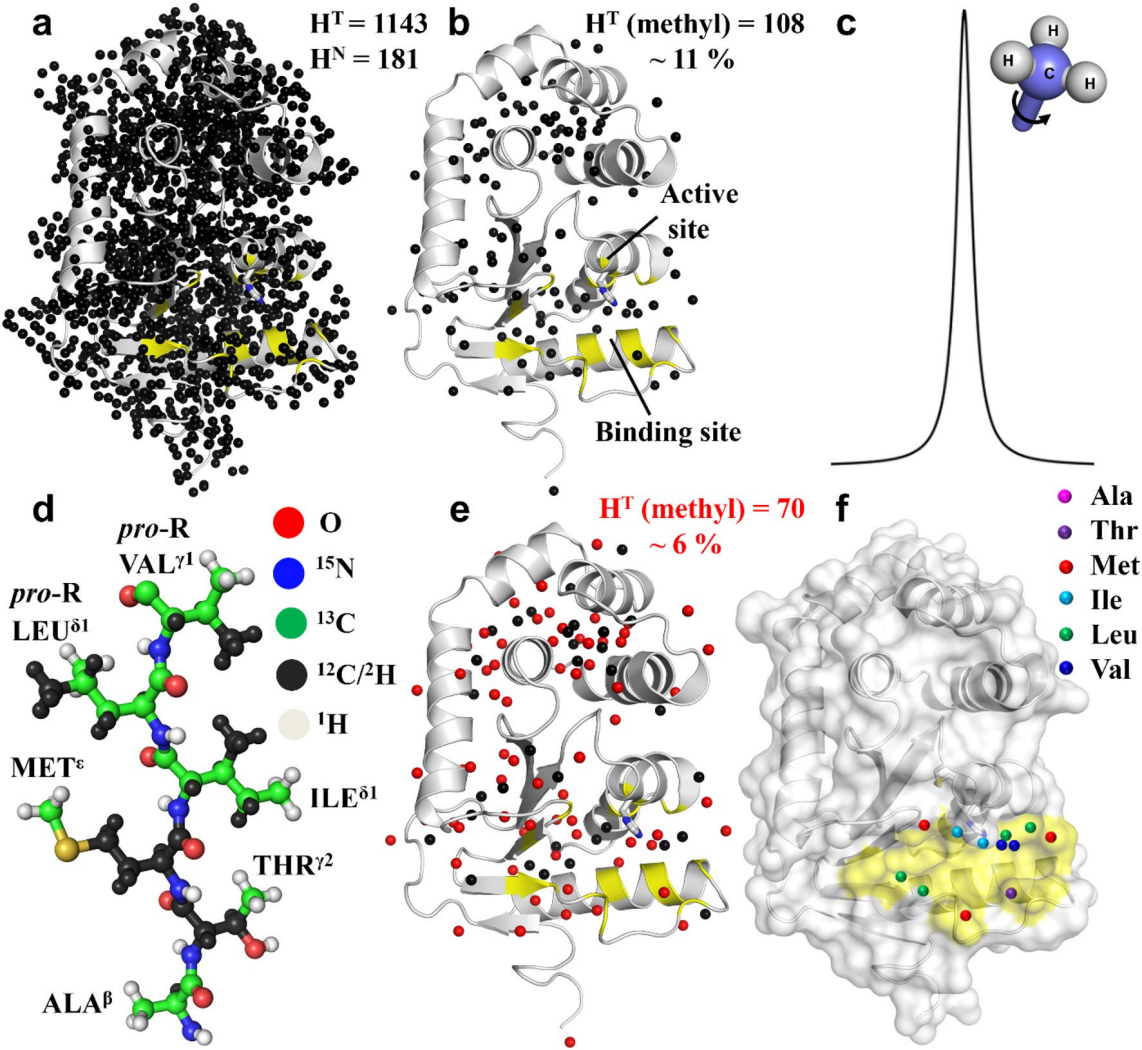
Labelling strategy used in the study. Schematic representation of the proton density in the structure of the bacterial oxidoreductase enzyme *Ec*DsbA, showing the position of amide (H^N^) and total (H^T^) protons (**a**) when fully protonated, (**b**) when fully deuterated except for methyl groups, which represent 11% of all protons. (**c**) Schematic showing that the fast rotation of methyl groups results in narrow linewidths in NMR spectra. (**d,e**) Labelling scheme used in the triple labelled sample where only one methyl group is protonated in Ile, Leu and Val, decreasing the proton density to 6% versus the fully protonated protein. (**e,f**) Residues that form the ligand binding site in *Ec*DsbA are colored yellow and the positions of methyl-containing residues adjacent to the binding site are highlighted. H^T^ indicates total number of proton resonances expected in the NMR spectrum of oxidized *Ec*DsbA (PDB ID: 1FVK). Total number of expected proton resonances was calculated assuming methyl/methylene/symmetric aromatic protons had the same chemical shift. Analysis was done by MOLMOL.

We generated structural models for the ligand bound to oxidized *Ec*DsbA using four different approaches: X-ray crystallography, *N*MR^2^, NMR data driven docking with HADDOCK^4^, and classical NMR structure calculation using CYANA. All four approaches found similar binding poses with small variation in the positioning of phenylthiazole **1** (*vide infra*). *Ec*DsbA was selectively labelled with protonation on the following methyl groups: A^β^I^δ1^(LV)^**proR**^M^ε^T^ϒ2^ while the remaining protons were deuterated^21,22^. Conventional NOESY experiments from [U-^2^H]-A^β^I^δ1^(LV)^proR^M^ε^T^ϒ2^-CH_3_ labelled EcDsbA and phenylthiazole **1** may provide ambiguous intermolecular NOE cross-peaks in the amide region due to peak overlap between amide resonances of the protein and aromatic resonances of the ligand, therefore, the NMR experiments were performed in D_2_O NMR buffers (see Supplementary Fig. S2 and S3). Labelling all methyl-containing residues provides greater coverage of the protein surface than selective methyl labelling approaches^23,24^. Moreover, the labelling pattern of the ILV methyl groups in the current scheme reduces the complexity of the NMR spectra because only one methyl group is labelled in each of these residue types, thereby providing unambiguous and stereospecific information (Fig. 1). In addition to reducing peak overlap, spin diffusion is also considerably reduced through this labelling scheme due to the lower proton density. Spin diffusion is a major limitation when measuring direct ^1^H-^1^H magnetization transfer as it dominates the NOE cross peak intensity at high mixing times. The direct NOE transfer can be converted into a meaningful distance, but the spin diffusion introduces uncertainty into the distance restraints.

**Figure 2:**
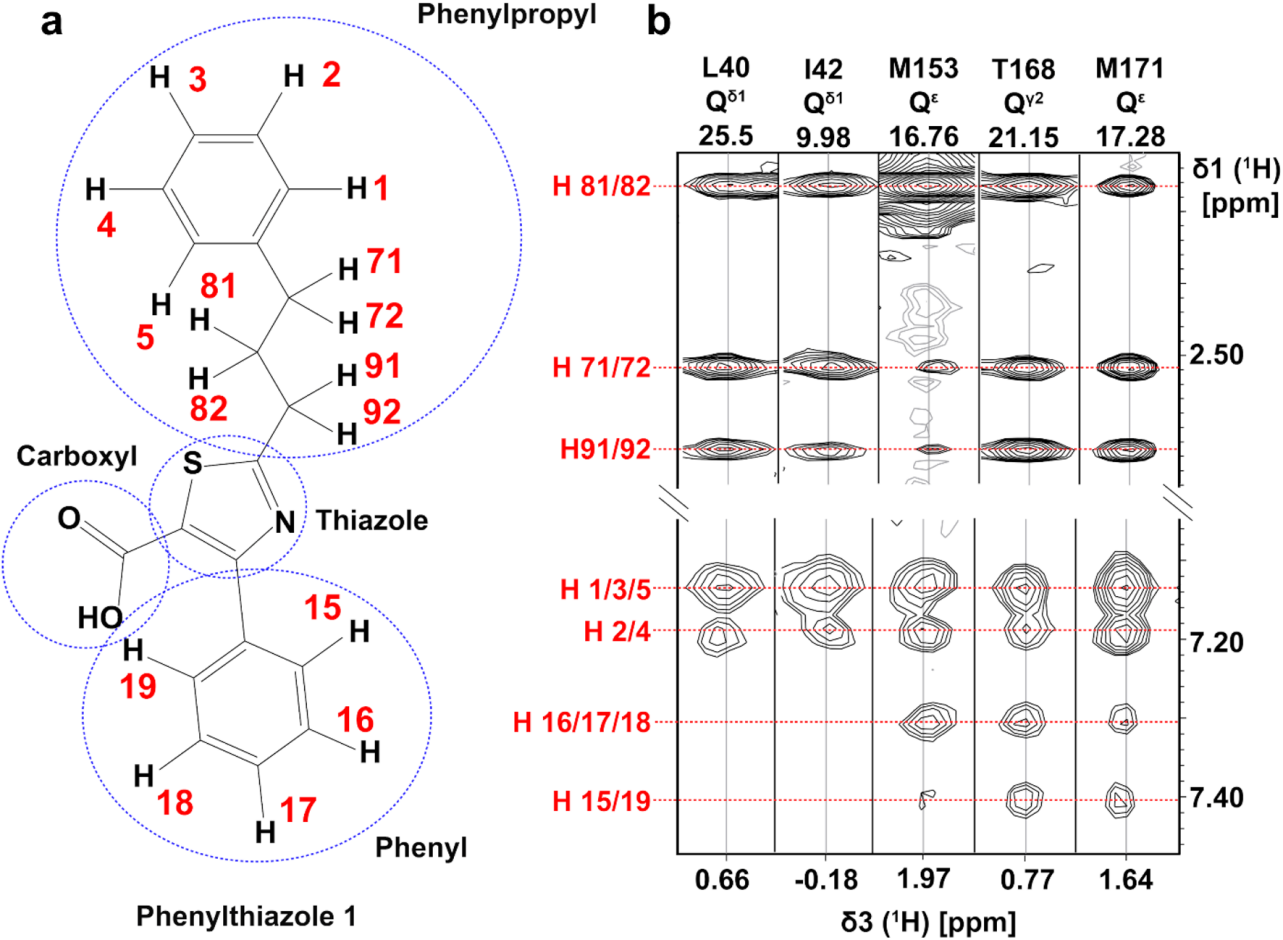
Intermolecular NOEs between oxidized EcDsbA and phenylthiazole **1**. (**a**) Chemical structure of phenylthiazole **1**. (**b**) [^1^H,^1^H] strips from 3D ^13^C-edited [^1^H,^1^H]-NOESY HMQC spectrum of 0.25 mM *Ec*DsbA with 3.5 mM phenylthiazole **1** recorded at 298 K with an NOE mixing time of 400 ms.

Replacing most of the protons in the protein with deuterons improves signal to noise and reduces spin-diffusion, which preserves the quality of the distance information contained in the NOE cross peaks. The NOE cross peaks are shown in Figure 2, and Supplementary Table S1 contains the corresponding estimated NOE distances observed in a sample containing 0.25 mM *Ec*DsbA and 3.5 mM phenylthiazole **1**. Measurement of ligand solubility revealed that phenylthiazole **1** was soluble and free from aggregation at this concentration^25^ (Supplementary Fig. S4). The ligand affinity (*K*_D_) was estimated at 0.9 mM based on chemical shift perturbations measured in HSQC spectra recorded with increasing ligand concentration. Consequently, 79% of *Ec*DsbA was in the ligand-bound state under the experimental conditions^26^ (Supplementary Fig. S5). For the HADDOCK protocol as well as for the classical NMR structure calculation, protein assignments were obtained using standard NMR experiments.

**Figure 3:**
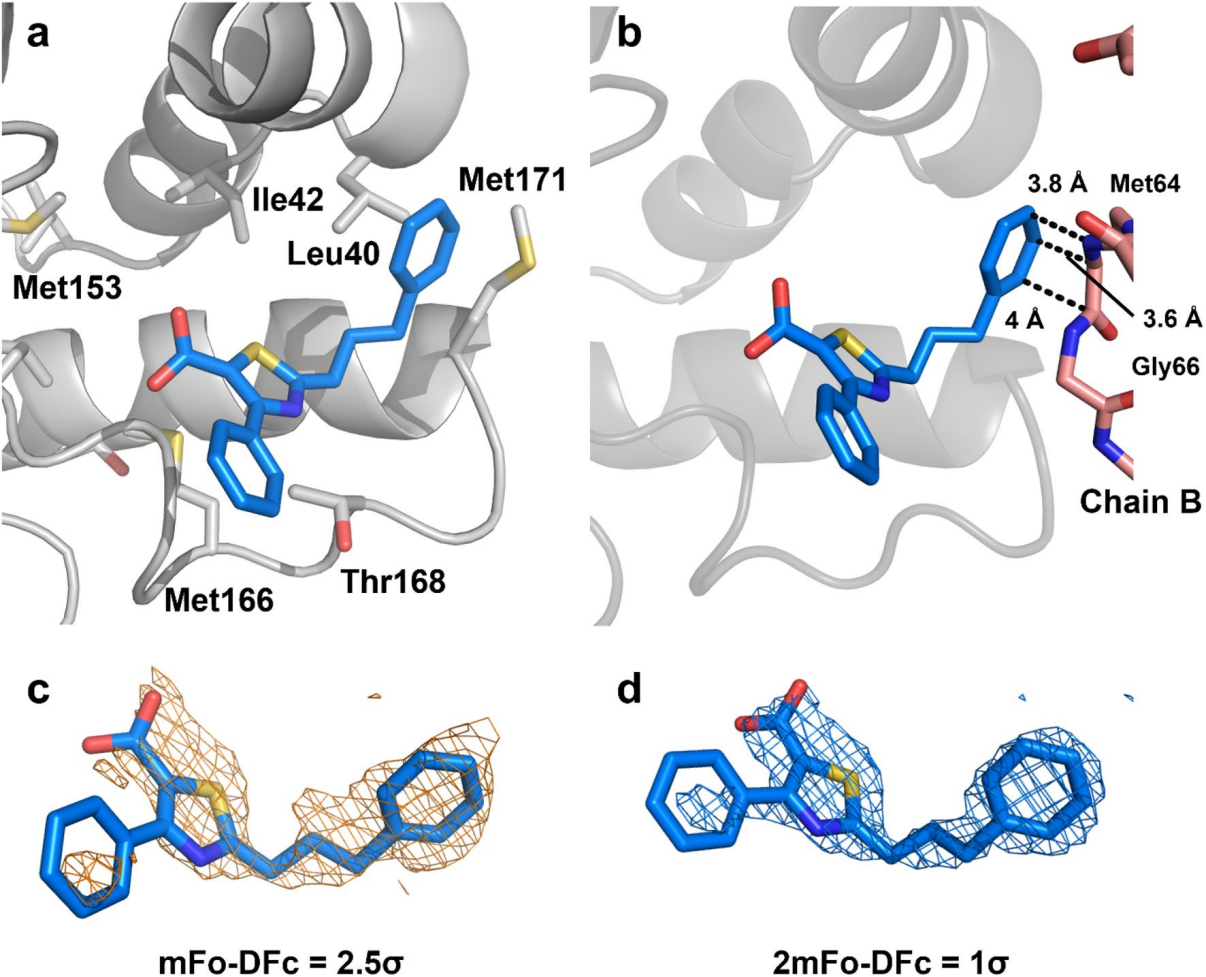
X-ray structure of *Ec*DsbA–phenylthiazole **1** complex at 2.0 Å resolution. (**a**) Ligand is shown in blue sticks and the protein is in grey cartoon. Several side chain residues of *Ec*DsbA at the binding site are shown in sticks. Only the binding site region is shown for clarity. (**b**) Crystal contacts between the heavy atoms of Chain B (depicted in salmon sticks) and the ligand are shown as dotted black lines. (**c**) Simulated annealing omit σ_A_-weighted mFo-DFc electron density map of phenylthiazole **1** contoured at 2.5σ and displayed in yellow mesh. (**d**) σ_A_-weighted 2mFo-DFc electron density map of phenylthiazole **1** contoured at 1σ and displayed in blue mesh.

An X-ray crystal structure of the complex was determined by soaking crystals of *Ec*DsbA with phenylthiazole **1** and refined to 2.0 Å of resolution (Fig. 3; see Supplementary Table S2). The crystals contained two protomers in the asymmetric unit and, as illustrated in Fig. 3, partial electron density was observed for only one phenylthiazole **1** molecule, which was bound to the hydrophobic groove of chain A, but with some additional contacts being made between the ligand and residues of chain B. The structures derived from *N*MR^2^ were calculated using the intermolecular protein–ligand NOE list without protein assignments (see Supplementary Table S1).

NOESY data were acquired with two mixing times to derive the distance restraints from the initial rates of the NOE build-up curves. NOE build-up curves that did not exhibit an initial linear behavior were not used in the analysis (see Supplementary Fig. S6). Two mixing times are the minimum required since these provide the three data points (two measured data points and the zero-time point) that are needed to check the initial linearity of the build-up curves. The diagonal peak decays were also evaluated because the initial magnetization and the auto-relaxation rates are needed to extract accurate distances^27^. The intermolecular NOEs were corrected for the protein occupancy since the ligand is a weak binder in fast exchange compared to the chemical shift and relaxation time scales. The distances obtained in this way are more accurate compared to traditional NOE-derived distances but are called semi-ambiguous due to the lack of protein assignment^28^. Nonetheless, the restraints are used differently compared to the ambiguous restraints previously defined in the literature^29,30^. The standard ambiguous restraint allows multiple protons, arising from two or more resonances, be used simultaneously to fulfil one NOE. In the *N*MR^2^ protocol, this is forbidden, and each NOE restraint must explicitly relate to two groups of protons, corresponding to two NMR resonances. This analysis provides more explicit distance information compared to the classical ambiguous restraints but requires that NOEs involving overlapping peaks cannot be used in the *N*MR^2^ calculation. To minimize the possibility of peak overlap a high-resolution constant time 2D [^13^C,^1^H]-HSQC spectrum was recorded. In combination with our labeling scheme this distinguishes between different residue types, and in this case excellent resolution was observed in the 3D carbon resolved NOESY-HMQC spectra (Supplementary Fig. S2, and Fig. 2). Our labelling scheme also avoids the use of isotope-filtered NMR experiments. This affords spectra with greater signal to noise due to the increased sensitivity of standard NOESY pulse sequences. To further increase resolution and sensitivity of intermolecular cross peaks in the indirect NOESY dimension, non-uniform sampling acquisition schemes can be used (see Supplementary Fig. S7). In addition to the protein–ligand NOEs, the non-assigned protein intra methyl-methyl distances derived from the 3D ^13^C-resolved NOESY-HMQC can also be used by *N*MR^2^ since the structure calculation protocol does not require the methyl assignments (see Supplementary Table S1).

**Figure 4:**
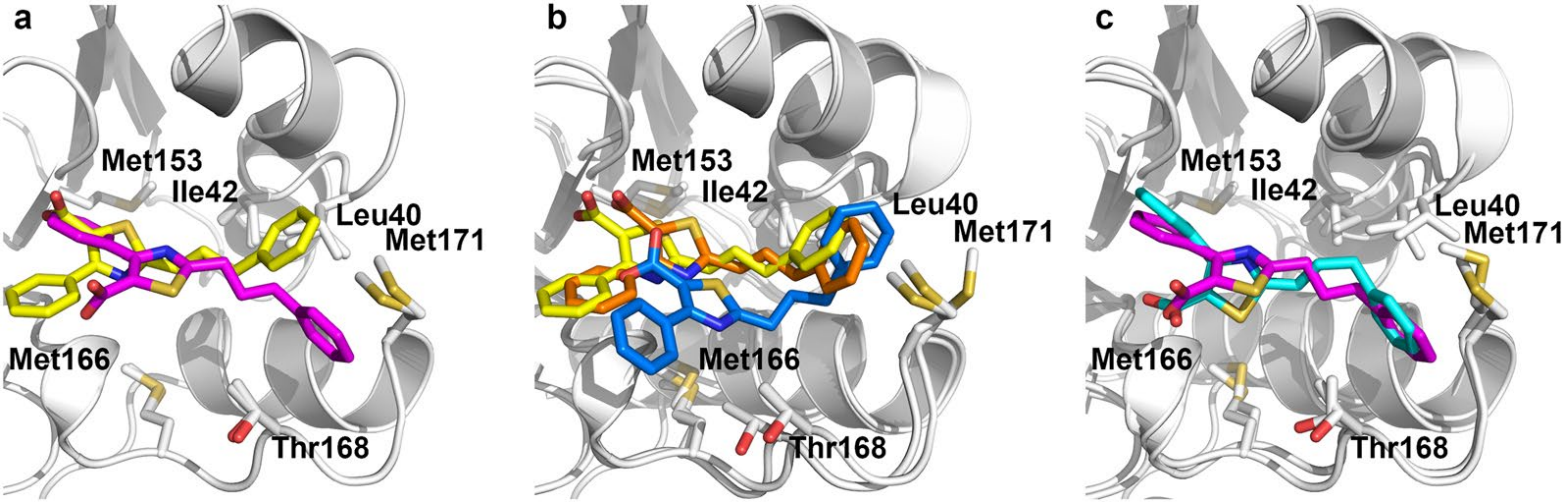
*Ec*DsbA*–*phenylthiazole **1** complex structures derived by *N*MR^2^, X-ray crystallography and classical NMR structure calculation. (**a**) Overlay of best and second-best *N*MR^2^ models depicted in yellow and magenta, respectively. (**b**) Comparison of best *N*MR^2^ model against classical CYANA and crystal structures, depicted in yellow, orange, and blue respectively. (**c**) Comparison of the second-best pose of *N*MR^2^ against the best HADDOCK model, depicted in cyan. All models were globally aligned to the crystal structure in PyMOL. Only the binding site of all models is shown for clarity.

For the *N*MR^2^ calculations, the bound conformation of the ligand was calculated using tr-NOEs derived from short 2D ω1, ω_2_-^13^C,^15^N-filtered [^1^H,^1^H]-NOESY spectra (data not shown)^3,31–33^. Using the top 20 bound conformations of the ligand, an input structure of the protein (PDB code 1FVK), the set of semi-ambiguous intermolecular distances, and the unassigned protein methyl-methyl distances, *N*MR^2^ was employed to determine the structure of phenylthiazole **1** in the binding site of *Ec*DsbA. Figure 4a shows the best and the second-best *N*MR^2^ models.

*N*MR^2^ provides both the structure of the complex and a partial assignment for the methyl groups. The assignments produced in this way may contain errors since *N*MR^2^ is designed to calculate the structures that best satisfy the experimental distance constraints. In cases where two methyl groups are near to one another, or two methyl groups are equidistant from a ligand proton, it is possible to swap the methyl assignments without impacting the structure. For example, in the structure of *Ec*DsbA bound to phenylthiazole **1**, the methyl protons of Met153 and Met166 are located at similar distances from the ligand. In this case their assignments can be swapped. This may account for the variation in the positioning of phenyl carboxylic acid group between two *N*MR^2^ models (Fig. 4a). However, the assignments for the other methyl resonances that were used in the *N*MR^2^ calculations were consistent with assignments obtained from conventional methods. Eleven high-quality NOE build-up curves were observed from the 3-phenylpropyl moiety to Leu40^δ1^, Ile42^δ1^, Thr168^γ2^ and Met171^ε^ and one NOE was observed between Met153^ε^ and the second phenyl moiety of phenylthiazole **1**. A comparison of intermolecular ^1^H-^1^H distances derived from the crystal structure and NOE data is shown in Supplementary Table S3. Structure calculations were also performed using two classical NMR-based approaches: HADDOCK and CYANA, where the protein resonance assignments were utilized. The classical structure calculation was performed with CYANA using the NOE constraints from Supplementary Table S1 with known methyl assignments. A model with lowest target function (TF) from the classical CYANA structure calculation is shown in Fig. 4b. The binding mode of phenylthiazole **1** from CYANA is similar to the best *N*MR^2^ model despite one difference in the methyl assignments. This discrepancy is not structurally relevant because the NOE derived distance involved in two different assignments (M5—Gln59 corresponds to Met153/M166-Q^ε^—Gln59 in Supplementary Table S1) is large and cannot discriminate between the two^34^. Furthermore, both NOE-derived distance restraints and CSP were used to generate constraints in calculating the structure of the complex using HADDOCK (see Supplementary Table S3). An ensemble of the top five lowest energy HADDOCK models is shown in Supplementary Fig. S8. A unique binding mode of phenylthiazole **1** was observed in all HADDOCK models which is similar to the second best *N*MR^2^ model. The best HADDOCK model (with the lowest HADDOCK score among the five models) is shown in Fig. 4c.

The four structural models derived from X-ray crystallography and NMR calculations all exhibit binding of phenylthiazole **1** to the same binding site on a shallow groove of oxidized *Ec*DsbA (Fig. 4a–c). In each case the 3-phenylpropyl moiety is located between the two α-helices that define the hydrophobic groove and close to Met171 while the second phenyl group is either pointing away from the protein, where it is more solvent exposed (Fig. 4b) or towards the protein (Fig. 4c). The 3-phenylpropyl moiety in all structures makes hydrophobic contacts to Leu40, Met171 and Ile42, while the second phenyl ring orients towards either Met166 (Fig. 4b) or Met153 (Fig. 4c). The thiazole ring also shares a similar location in the binding site but with different orientations of the carboxylate group. The X-ray structure, the best *N*MR^2^ and the CYANA models have the carboxylate oriented towards the protein, whereas the second best *N*MR^2^, and the HADDOCK models orient the carboxylate towards the solvent. In addition, where the carboxylic acid group is oriented towards the protein, it binds deeper into the binding site in the NMR models compared to the X-ray structure. This discrepancy is difficult to interpret due in part to the fact that only partial electron density was observed for phenylthiazole **1** in the crystal structure (Fig. 3). We hypothesize that the binding mode in which the carboxylate is buried and the phenyl ring points towards the solvent is less likely in solution (Fig. 4b) because no intermolecular NOEs were observed between phenylthiazole **1**-H15/H19 and Thr168-Q^γ2^, which is what would be expected from the orientation observed in the X-ray crystallography structure (see Supplementary Table S3). In addition, several crystal contacts between the ligand and a neighbouring protein are observed and could explain the discrepancies between the NMR and X-ray structures (Fig. 3b). While the classical NMR models are similar to the *N*MR^2^ structures, the time taken to generate models of the complex structure is orders of magnitude greater due to the requirement that sequence specific assignment of the resonances must be obtained. In essence, the *N*MR^2^, classical CYANA, HADDOCK and X-ray structures are all reporting binding at the same position, which was proposed to be the substrate-binding site of *Ec*DsbA^35^. In each case the model indicates that the phenylthiazole core binds in a similar orientation along the hydrophobic groove, while the largest differences in the structural models are located at the solvent-exposed groups. Essentially, *N*MR^2^ generates models in a reasonable time frame, which are sufficient to define vectors on the fragment molecules where the fragment can be further expanded or optimized through medicinal chemistry. *N*MR^2^ does not overcome the challenges that result from the low affinity of the ligand, and so relatively high protein concentrations are required to afford the necessary sensitivity. Nonetheless, suitable amounts of the selectively labelled protein sample can be produced to run the experiments successfully.

In summary, we have shown that selective labeling of all methyl groups of a protein in a deuterated background yields useful probes for utilizing *N*MR^2^ in the process of structure-based drug design with large complexes. We also report the binding mode of phenylthiazole **1** at the substrate binding site of *Ec*DsbA. We anticipate a broad application of the proposed *N*MR^2^ spectroscopy approach in the future of medicinal chemistry projects that are not amenable to X-ray crystallography.

## Methods

### Protein expression and purification of selective methyl labelled sample

*E. coli* BL21 (DE3) cells carrying the plasmid B0013-(5644bb) coding for *Ec*DsbA were first adapted to D_2_O by repeated subculturing with an increasing percentage of D_2_O in the medium followed by double colony-selection^36^. The colony showing the strongest growth was selected for preparation of glycerol stocks, which were stored at −80 °C. To produce a labelling pattern of A^β^I^δ1^(LV)^proR^M^ε^T^ϒ2^-^13^CH_3_ in a deuterated background (hereafter referred to as [U-^2^H]-A^β^I^δ1^(LV)^proR^M^ε^T^ϒ2^-^13^CH_3_), we used a HLAM-A^β^/I^δ1^/LV^*proR*^, U-^13^C, M^ε^/T^γ^ kit from NMR-Bio (http://www.nmr-bio.com/) according to the manufacturer’s instructions. In this labelling scheme, the alanine β, isoleucine δ1, methionine ε, and threonine γ methyl positions are selectively protonated. The methyl groups of leucine and valine residues are stereospecifically labelled at pro-R positions. The methionine and threonine methyls are selectively ^13^C-labelled, with the remainder of the side chain being ^12^C. Alanine, isoleucine, leucine and valine residues are labelled with ^13^C as depicted to generate a linear spin system (Fig. 1d). ^15^NH_4_Cl was added into the expression media to allow for ^15^N incorporation at each amino acid site. To suppress scrambling from threonine to glycine, unlabeled glycine was also included in the expression media. As a result, glycine backbone amide resonances are weak in the [^15^N,^1^H]-HSQC. The methionine and threonine precursors did not contain ^15^N so the weak cross-peaks for methionine and threonine residues in the [^15^N,^1^H]-HSQC are possibly due to a transamination effect (Supplementary Fig. 3a). Pre-expression cultures were prepared from 10 mL M9/D_2_O medium supplemented with 1 g/L ^15^NH_4_Cl and 2 g/L D-glucose-^13^C_6_-1,2,3,4,5,6,6-d_7_, inoculated from the glycerol stock and incubated for 16 h at 37 °C with constant agitation at 200 rpm. ^15^NH_4_Cl was added into the expression media to assess the efficiency of ^15^N incorporation at each amino acid site. 250 mL M9/D_2_O medium supplemented with 1 g/L ^15^NH_4_Cl and 2 g/L D-glucose-^13^C_6_-1,2,3,4,5,6,6-d_7_ was inoculated with 5 mL pre-expression culture and incubated at 37 °C with constant agitation at 200 rpm. The D_2_O solutions of 2-(D_3_)-methyl-1,2,3,4-(^13^C_4_)-acetolactate and 2,3,3,4,4-(D_5_)-(^13^C)-methyl-l-methionine were added to the culture 1 h before induction (OD_600_ ~ 0.6). 15 min prior to induction, the D_2_O solutions of 2-hydroxy-2-[2’-(^13^C),1’-(D_2_)]-ethyl-3-oxo-4-(D_3_)-butanoic acid, U-(^13^C)-2-(D)-l-alanine, 2,3-(D_2_),4-(^13^C)-l-threonine and deuterated glycine were added. When the optical density at 600 nm of the growth culture reached 0.8, the culture was induced with 1 mM IPTG and incubated for 16 h at 18 °C with constant agitation at 200 rpm. The cells were harvested at 3200×*g* at 4 °C for 20 min and stored at −80 °C. The protein was subsequently purified and oxidized as previously described^37^. To achieve complete backbone amide deuteron-to-proton exchange, the protein was partially unfolded in 2 M guanidine hydrochloride for 1 h at room temperature and refolded by rapid dilution in a tenfold volume of buffer (50 mM HEPES (pH 6.8), 50 mM NaCl). After a further desalting step, the refolding yield was approximately 70%. The total cost of the selective methyl labelling precursors used in preparation of the sample was ~ €400, which yielded ~ 1.6 mg of refolded protein.

### Binding affinity of phenylthiazole 1 and oxidized *Ec*DsbA

To determine the equilibrium dissociation constant (*K*_D_) for phenylthiazole **1** binding to *Ec*DsbA, [U-^15^N]-labelled oxidized *Ec*DsbA was prepared in HEPES buffer (50 mM HEPES (pH 6.8), 50 mM NaCl) with 90% H_2_O and 10% D_2_O. A series of NMR samples (each at a total volume of 160 µL) was prepared containing 50 µM protein and different concentrations of phenylthiazole **1** and transferred into 3 mm thin-walled NMR tubes. d_6_-DMSO concentration was 2% (*v/v*) in each sample. For NOESY experiments, 350 µL of 0.25 mM [U-^2^H]-A^β^I^δ1^(LV)^proR^M^ε^T^ϒ2^-^13^CH_3_-labelled oxidized *Ec*DsbA and 3.5 mM unlabeled phenylthiazole **1** was prepared in 50 mM NaPi (pH 6.8), 25 mM NaCl, with 98% D_2_O and 2% d_6_-DMSO and transferred to a Shigemi NMR tube. The reported pH of buffers in D_2_O are uncorrected meter reading. Spectra were processed using Topspin. Peak assignments in the individual spectra were manually adjusted by CARA. Chemical shift perturbations (CSP) were calculated using the equation as described previously^6^. *K*_D_ was calculated with GraphPad Prism© using the ligand depletion model and the equation as previously described.

### NMR data acquisition, processing and data analysis

All NMR spectra were collected at 298 K on Bruker 600 MHz or 800 MHz spectrometers equipped with CryoProbes. For tracking side-chain methyl CSP in the presence of phenylthiazole **1**, constant time [^13^C,^1^H]-HSQC spectra of [U-^2^H]-A^β^I^δ1^(LV)^proR^M^ε^T^ϒ2^-^13^CH_3_-labelled *Ec*DsbA with and without phenylthiazole **1** were collected. 3D ^13^C_ali_-edited [^1^H,^1^H]-NOESY-HMQC (NOE mixing times of 100 ms and 400 ms) experiments of 0.25 mM [U-^2^H]-A^β^I^δ1^(LV)^proR^M^ε^T^ϒ2^-^13^CH_3_-labelled *Ec*DsbA and 3.5 mM phenylthiazole **1** were acquired, either by uniform sampling or by non-uniform samples drawn from a probability density function with an exponential decay^38^. To acquire 3D NUS ^13^C^methyl^-edited [^1^H,^1^H]-NOESY-HMQC, 25% of 30 × 512 complex points were acquired for the indirect carbon and NOESY dimensions respectively in ~ 27 h. 16 transients per FID were acquired and NOE mixing time was set to 400 ms. Uniformly sampled 3D ^13^C^methyl^-edited [^1^H,^1^H]-NOESY-HMQC data were collected with the same acquisition parameters but with 8 transients per FID in 54 h. Uniformly sampled NOESY data were processed by Topspin. A series of 2D ω1, ω2-^13^C,^15^N-filtered [^1^H,^1^H]-NOESY spectra was collected with NOE mixing of 10 ms, 20 ms, 40 ms and 70 ms, to calculate the bioactive ligand conformation in solution. Linearly sampled datasets were processed using Topspin 3.2 (pl6) and non-uniformly sampled datasets were processed either by compressed sensing in Topspin or using hmsIST^39^. The spectra were converted to XEASY format for analysis in CARA. Previously obtained backbone and side-chain methyl assignments were used as the input of CARA however prochiral methyl assignments of Leucine and Valine residues were established from the constant time [^13^C,^1^H]-HSQC spectrum of [U-^2^H]-A^β^I^δ1^(LV)^proR^M^ε^T^ϒ2^-^13^CH_3_-labelled *Ec*DsbA^23^.

The methyl-specific isotope labelling used here allows the assignment of the type of amino acid residue based on the chemical shift of the ^13^C methyl resonance. Moreover, because of the labelling strategy, it is possible to distinguish between residue types depending on whether the ^13^C-methyl carbon is directly attached to another ^13^C atom or not (Fig. 1). In the case of methionine, the methyl carbon is attached to a sulfur atom and for threonine the methyl is attached to a ^12^C^β^ carbon. This results in peaks in the constant time HSQC spectrum having different phases, with the negative peaks belonging to the methyl group resonances of the threonine and the methionine (see Supplementary Fig. S2) and the positive peaks corresponding to the other methyl resonances. Methyl peaks corresponding to the amino acid residue types Met and Thr can therefore directly be identified by their phase and chemical shift and this assignment information can be used by *N*MR^2^.

### Solubility of phenylthiazole 1 in D_2_O NMR buffer

A 2D [^1^H,^1^H]-NOESY spectrum at 4 mM phenylthiazole **1** in D_2_O NMR buffer (50 mM NaPi (pH 6.8), 25 mM NaCl, 2% d_6_-DMSO) was acquired at 298 K. The NOE mixing time was set to 800 ms. The NOE cross peak signals are of opposite sign to the diagonal peaks, suggesting the ligand is soluble at a concentration of 4 mM (see Supplementary Fig. S4).

### Proton chemical shift assignments of phenylthiazole 1 in its free and bound states

Proton chemical shifts of free phenylthiazole **1** in D_2_O NMR buffer (phenylthiazole **1** concentration 4 mM; 50 mM NaPi (pH 6.8), 25 mM NaCl, 2% d_6_-DMSO) were assigned using 1D ^1^H, 2D [^13^C,^1^H]-HMBC and 2D [^1^H,^1^H]-NOESY spectra. ^1^H chemical shift assignments of phenylthiazole **1** in the protein bound state were made using 1D ^1^H and 2D ω1, ω2-^13^C,^15^N-filtered [^1^H,^1^H]-NOESY spectra.

### NMR structure calculation with HADDOCK

The data-driven HADDOCK docking calculation was performed using the protocol as described previously by Mohanty et al.^23^. The topology and parameter files of phenylthiazole **1** for HADDOCK were created using ATB server^40^. 40 protein conformers sampled from both chain A and B (PDB ID:1FVK) were provided as the input of HADDOCK. To account for ligand flexibility, 26 discrete sets of ligand conformers were generated using the ConfGen advanced panel in Schrödinger Maestro. The residues that exhibited CSP > 0.02 ppm and had a calculated solvent accessibility above 15% were selected for use as ambiguous intermolecular distances in HADDOCK. *N*MR^2^ input NOE lists with protein assignments were used for HADDOCK. The intermolecular NOE distance constraints were tabulated in CNS format with the lower distance limit (target distance in Å – 1.8 Å) and the upper distance limit (target distance in Å + 15% of target distance in Å) as described^23^. 10,000 models were generated in it0 (rigid body docking stage) and 400 models were calculated in it1 (semi-flexible simulated annealing) followed by water refinement. Of the final 400 water refined HADDOCK models, the 10 lowest energy structures with minimal distance restraint violations (< 0.5 Å) were selected for the analysis. The electrostatic flag was switched on during the HADDOCK calculation. The segment “Q164-D172” was defined as fully flexible throughout HADDOCK docking as reported previously^23^.

### X-ray structure determination

Soaking of phenylthiazole **1** into *Ec*DsbA crystals was performed using the protocol as described previously^37^. *Ec*DsbA*–*phenylthiazole **1** complex structure coordinates have been deposited into PDB with accession number 7TTV.

### *N*MR^2^ structure determination

The *N*MR^2^ method does not use any force field to calculate the structures, while the structures derived from HADDOCK and X-ray crystallography refinement software are optimized with explicit water molecules. All spectra were processed with Topspin 3.1 (Bruker). The spectra were evaluated with ccpNMR analysis 2.3^41^. Distances were derived from NOE build-up curves using a simple two-spin system model (i,j) and following the established protocol (see Supplementary Table S1)^3,15,28,42,43^. The auto-relaxation rates, *ρ*_*i*_, and initial magnetizations, *ΔM*_*ii*_(*0*), were extracted using a mono-exponential decay function, *ΔM*_*ii*_(*t*) = *ΔM*_*ii*_(*0*) exp(–*ρ*_*i*_*t*). Missing auto-relaxation rates were set to the median of the experimentally derived ones. Cross-relaxation rates, *σ*_*ij*_, were fitted using a two-spin system approximation model for the protein–ligand NOEs, *ΔM*_*ij*_(*t*), Eq. (1)^44^. The corresponding distances, *r*_*ij*_, are derived from the cross-relaxation rates, *σ*_*ij*_, defined in Eq. (3),

1 $$\frac{{\Delta M_{ij} (t)}}{{\Delta M_{ii} (0)}} = - \frac{{\sigma_{ij} }}{{\lambda_{ + } - \lambda_{ - } }}(e^{{ - \lambda_{ - } t}} - e^{{ - \lambda_{ + } t}} )$$

2 $$\lambda_{ \pm } = \frac{{\rho_{i} + \rho_{j} }}{2} \pm \sqrt {\left( {\frac{{\rho_{i} - \rho_{j} }}{2}} \right)}^{2} + \sigma_{ij}^{2}$$

3 $$\sigma_{ij} = \frac{{b^{2} }}{{r_{ij}^{6} }}(6J(2\omega ) - J(0))$$

4 $$J(\omega ) = \frac{2}{5}\left( {\frac{{\tau_{{\text{c}}} }}{{1 + (\omega \tau_{{\text{c}}} )^{2} }}} \right)$$

5 $$b = \frac{1}{2}\frac{{\mu_{0} }}{4\pi }\hbar \gamma_{{\text{H}}}^{2}$$

where *μ*_*0*_ is the permeability of free space, *ħ* the reduced Planck constant and γ_H_ the gyromagnetic ratio of the nucleus and *τ*_*c*_ the rotational correlation time of the protein–ligand complex. 37 intermolecular and 49 intra-ligand NOEs could be measured. Excluding build-up curves with large spin diffusion contributions, Supplementary Fig. S6 reduced the amount of distances restraints to 12 for the protein–ligand and 19 for the ligand alone. Because the bound conformation of the ligand was poorly converged, we used the 20 best ligand conformations as input for the *N*MR^2^ calculations. Then the ligand conformation was restrained during the *N*MR^2^ calculations while the protein side chains were allowed to adjust. The *N*MR^2^ structure is the one with the lowest CYANA target function (target function ~ 1.0 Å^2^). As expected from the NOE data set, the *N*MR^2^ second-best binding mode (target function ~ 1.5 Å^2^) is the mirror image along the long axis of the ligand of the best structure. This is due to the symmetry of the NMR restraints from the ligand aromatic rings. The structures were then minimized (100 steepest descent minimization steps) in Chimera using the amberff14sb force field and Gasteiger partial charges for the ligand.

## Supplementary Information

Supplementary Information.

## Data Availability

The datasets and analysis generated in the current study are available upon request (correspondence to martin.scanlon@monash.edu and julien.orts@univie.ac.at).
