# Supplementary material for: Methyl probes in proteins for determining ligand binding mode in weak protein–ligand complexes
Source: Sci Rep. 2022 Jul 4;12:11231. doi: 10.1038/s41598-022-13561-y (PMC9253027; doi:10.1038/s41598-022-13561-y)

Supplementary Information

Methyl probes in proteins for determining ligand binding mode in weak protein*–*ligand complexes

# Biswaranjan Mohanty1,2,3,+, Julien Orts4,+ ,*, Geqing Wang5, Stefan Nebl1, Wesam S. Alwan1, Bradley C. Doak1,2, Martin L. Williams1, Begoña Heras5, Mehdi Mobli6 and Martin J. Scanlon1,2,*

1Medicinal Chemistry, Monash Institute of Pharmaceutical Sciences, Monash University, 381 Royal Parade, Parkville, 3052, Victoria, Australia

2ARC Centre for Fragment-Based Design, Monash Institute of Pharmaceutical Sciences, Monash University, 381 Royal Parade, Parkville, 3052, Victoria, Australia

3Sydney Analytical Core Research Facility, The University of Sydney, Sydney, New South Wales 2006, Australia

4University of Vienna, Department of Pharmaceutical Sciences, Althanstrasse 14, A-1090 Vienna, Austria

5La Trobe Institute for Molecular Science, La Trobe University, Melbourne, VIC, 3085, Australia

6Centre for Advanced Imaging, The University of Queensland, St Lucia, 4072, Queensland, Australia

*Correspondence and requests for materials should be addressed to [martin.scanlon@monash.edu](mailto:martin.scanlon@monash.edu) and [julien.orts@univie.ac.at](mailto:julien.orts@univie.ac.at)

+these authors contributed equally to this work

Supplementary tables and figure legends

**Table S1**. **NOE-derived interproton distances.** Intermolecular *Ec*DsbA*–*phenylthiazole **1** distances and intramolecular *Ec*DsbA*–Ec*DsbA distances were derived from the 3D ^13^C edited [^1^H,^1^H]-NOESY-HMQC spectra used to calculate the *N*MR^2^ structure. Only distances derived from build-up curves apparently free of spin diffusion and following a simple two-spin system model (Supplementary Fig. S6) were used. The distances are modified to account for proton multiplicity according to the CYANA specifications regarding the upper and lower limit distance restraints. [**a**] Phenylthiazole **1** proton assignments follow the conventions in Fig. 2, and methyl assignments of *Ec*DsbA are unknown and named as M1-M5. For degenerate protons in phenylthiazole **1**, we used “dummy” atoms and included the NOE contributions from all atoms that are defined by the dummy atom. For example, M1—Q9 contains the NOE contributions from H91 with M1 and H92 with M1. The assignment of the methyl resonances found in the best *N*MR^2^ structure is M1 = Thr168 QG2, M2 = Ile42 QD1, M3 = Met171 QE, M4 = Leu40 QD1, M5 = Met153 QE.

**Table S2. X-ray crystallography data collection and refinement statistics.**

^a^Values in parentheses refer to the highest resolution shell.

^b^Agreement between intensities of repeated measurements of the same reflections can be defined as:

$R_{merge}= \frac{\sum_{hkl} \sum_{i=1}^{n} \left| I_{i}\left( hkl \right)- \overline{I}(hkl) \right|}{\sum_{hkl} \sum_{i=1}^{n} I_{i}\left( hkl \right)}$

where *I_i_ (hkl)* are individual values and $\overline{I}(hkl)$ is the mean value of the intensity of reflection *hkl*.

^c^R_fac_ = ∑_h_ |F_o_ - F_c_| / ∑_h_|F_o_|, where F_o_ and F_c_ are the observed and calculated structure-factor amplitudes for each reflection “h”.

^d^R_free_ was calculated with 5% of the diffraction data selected randomly and excluded from refinement.

**Table S3.** Comparison of methyl-to-ligand ^1^H-^1^H distances derived from the crystal structure and NOE data. [**a**] Upper distance limits in Å [**b**] Not determined from NMR data [**c**] ^1^H-^1^H distances (Å) were computed between the pseudo hydrogen atom on a methyl group of *Ec*DsbA and individual hydrogen atom of phenylthiazole **1**. Pseudo atoms were added into the crystal structure of *Ec*DsbA by MOLMOL before estimating the distances in PyMOL.

**Table S4.** **Pairwise** **RMSD (Å) calculation between all models.** All models were superimposed (global fit by taking all atoms) in Maestro prior to the RMSD calculation.

**Figure S1.** ***N*MR^2^ structure determination workflow.** Workflow showing the different steps of *N*MR^2^ structure determination of protein–ligand complexes using A^β^I^δ1^(LV)^proR^M^ε^T^ϒ2^-^13^CH_3_ labelling protein sample in a deuterated background.

**Figure S2.** **2D constant time (ct) [^13^C,^1^H]-HSQC spectra from of [U-^2^H]-A^β^I^δ1^(LV)^proR^M^ε^T^ϒ2^-^13^CH_3_ labelled sample using NMR-BIO precursors.** Overlay of ct-[^13^C,^1^H]-HSQC spectra from of [U-^2^H]-A^β^I^δ1^(LV)^proR^M^ε^T^ϒ2^-^13^CH_3_-labelled oxidized *Ec*DsbA in presence (red/cyan) and absence (blue/magenta) of phenylthiazole **1**. Threonine and methionine methyl resonances have opposite phase in the ct-[^13^C,^1^H]-HSQC spectrum as they are not connected to any other ^13^C atom. Both spectra were collected in D_2_O NMR buffer (20 mM NaPi (pH 6.8), 50 mM NaCl) at 298 K on a 600 MHz spectrometer equipped with CryoProbe.

**Figure S3. H-D exchange analysis of oxidized *Ec*DsbA.** Conventional NOESY spectra from [U-^2^H]-A^β^I^δ1^(LV)^proR^M^ε^T^ϒ2^-CH_3_ labelled protein-ligand mixtures may provide ambiguous intermolecular NOE cross-peaks in the amide region due to peak overlap with aromatic ligand signals that usually occur in a similar region of the spectrum. Therefore, the protein sample was buffer exchanged from H_2_O (50 mM HEPES (pH 6.8), 50 mM NaCl) to D_2_O (20 mM NaPi (pH 6.8, 50 mM NaCl) NMR buffer. (**a**) [^15^N,^1^H]-HSQC spectra of oxidized *Ec*DsbA in H_2_O (50 mM HEPES (pH 6.8), 50 mM NaCl) NMR buffer. (**b**) [^15^N,^1^H]-HSQC spectra of oxidized *Ec*DsbA in D_2_O NMR in presence (red) and absence (blue) of phenylthiazole **1** prior to collecting intermolecular NOEs**.** (**c**) Heat-map of peak intensity of residual proteo amide signals mapped onto the crystal structure of *Ec*DsbA, showing that almost all the backbone amides in the binding site were replaced by deuterium. Color code: red – residues with residual proteo amide signals in [^15^N,^1^H]-HSQC; white – no signals detected; cyan – overlapped residues.

**Figure S4.** **Assignments and solubility of phenylthiazole 1 in D_2_O NMR buffer. (a)** ^1^H chemical shift assignments of phenylthiazole **1** in D_2_O buffer (50 mM NaPi (pH 6.8), 25 mM NaCl, 2% d_6_-DMSO) using 2D [^13^C, ^1^H]-HMBC. Vertical dashed lines in the aromatic region of HMBC indicate the multiple bond carbon to hydrogen connectivity. (**b**) Chemical structure of phenylthiazole **1.** Hydrogen atoms are numbered in red. Three bond coupling from 15/19 aromatic protons to the carbon atom in thiazole ring is highlighted. (**c**) Proton chemical shift assignments of phenylthiazole **1** in the complex were assigned by comparing the 1D ^1^H spectra of the free ligand (upper panel) and protein-ligand complex samples (lower panel). All the acquisition and processing parameters in both experiments were the same. The spectra were collected at 298 K on a Bruker AVANCE III 600 MHz spectrometer equipped with CryoProbe. (**d**) 2D [^1^H,^1^H]-NOESY spectrum of phenylthiazole **1**. NOE cross peaks are in opposite phase of the diagonal in 2D [^1^H, ^1^H]-NOESY suggesting that the ligand is soluble at 4 mM in the above D_2_O buffer. NOE mixing time of 800 ms was set to acquire NOESY spectrum.

**Figure S5.** **Chemical shift perturbations of [U-^15^N]-labelled oxidized *Ec*DsbA in the presence of unlabeled phenylthiazole 1**. *Ec*DsbA chemical shift perturbations (CSP), measured from amide groups, by successive phenylthiazole **1** titration ranging from 0.25 mM to 3 mM against 50 μM *Ec*DsbA. (**a-b**) [^15^N,^1^H]-HSQCs overlay of *Ec*DsbA without (blue) and with 3 mM phenylthiazole **1** (red)**.** (**c**) CSP histogram upon addition of 3 mM phenylthiazole **1** plotted against residue number. (**d**) Mapping CSP of phenylthiazole **1** (3 mM) to the structure of *Ec*DsbA, shown as color gradient in red (CSP ≥ 0.1 ppm) to white (CSP = 0 ppm). (**e**) Dissociation constant determination using the weighted CSPs of the amide resonances upon phenylthiazole **1** titration.

**Figure S6.** **NOE build-ups for the intermolecular NOE cross-peaks between oxidized *Ec*DsbA and phenylthiazole 1**. The cross-peak intensities were calculated from 3D ^13^C edited [^1^H,^1^H]-NOESY-HMQC spectra with NOE mixing times of 100 ms and 400 ms. The NOESY spectra were collected on a Bruker 800 AVANCE II spectrometer at 298 K. Acquisition and processing parameters were set the same in both cases. The build-up curves were fitted using the equation (1). The build-up curve from (**a**) does not follow equation (1) and was therefore excluded from the NMR structure calculations (*N*MR^2^, CYANA and HADDOCK), while the build-up curve (**b**) was consistent with equation (1) and was therefore included in the calculations.

**Figure S7. Comparison of intermolecular NOE cross peak intensities between uniform sampled versus non uniformly sampled (NUS) 3D NOESY.** (**a-b**) Two dimensional [^1^H,^1^H]-NOESY strips from 3D NUS ^13^C^methyl^-edited [^1^H,^1^H]-NOESY-HMQC spectra, acquired using noesyhmqcpr3d Bruker pulse sequence and reconstructed either by compressed sensing in Topspin (**a**) or hmsIST algorithms (**b**). (**c**) Chemical structure of phenylthiazole **1**. Hydrogen atoms are numbered in red. (**d**) Comparisons of intermolecular NOE cross peak intensities between uniformly sampled (US) Fourier transform processed spectrum versus non-uniform sampled compressed sensing (CS) and hmsIST-based reconstructed spectra. In our analysis, hmsIST found to be a better reconstruction method over CS as the hmsIST-reconstructed NOE cross peaks position and intensity are comparable to the US processed spectrum, indicating that the time saving due to NUS could be used to enhance spectral resolution whenever necessary.

**Figure S8. Lowest energy models derived by HADDOCK of the *Ec*DsbA*–*phenylthiazole 1 complex**. All 400 HADDOCK models were found to have similar binding modes. Five models are shown for clarity. The phenylthiazole **1** molecules are depicted as sticks in cyan. The oxygen atoms are depicted in red, the sulfur atoms in yellow and the nitrogen atoms in blue. Protein is represented with white ribbons and sticks following the same conventions.

Supplementary Table 1

| Protons^[a]^ | Distances [Å] |
| --- | --- |
| M1—Q9  M1—Q7  M2—Q9  M2—Q7  M2—Q15  M3—Q9  M3—Q7  M3—Q24  M3—Q15  M4—Q9  M4—Q7  M5—Q59  M1—M3  M2—M3  M2—M4  M2—M5  M3—M4 | 3.0—3.4  3.3—3.6  3.5—3.9  2.9—3.3  3.3—3.7  3.5—3.9  3.3—3.7  3.9—4.3  3.5—3.9  3.1—3.5  2.7—3.1  5.0—5.6  3.5—3.9  3.5—3.9  2.7—3.0  3.3—3.6  3.4—3.7 |

| **Supplementary Table 2**   \| Data collection \| Phenylthiazole **1** \| \| --- \| --- \| \| PDB ID \| 7TTV \| \| Space Group \| *C*2 \| \| Cell dimensions (Å) (a,b,c) \| (117.45, 63.74, 74.28) \| \| Angles \| α=γ=90° β=125.87° \| \| Resolution (Å)^a^ \| 37.08 - 1.99 (2.06 – 1.99) \| \| Total number of observations \| 273754 (26458) \| \| Number of unique observations \| 30612 (3010) \| \| Multiplicity \| 8.9 (8.8) \| \| Data Completeness (%) \| 99.71 (98.95) \| \| <I/σ_I_> \| 42.79 (4.76) \| \| *R_merge_*^b^ \| 0.7439 (1.235) \| \| Refinement \|  \| \| Resolution (Å) \| 37.08 - 1.99 (2.06 – 1.99) \| \| No. reflections \| 30557 (3009) \| \| *R*_work_^c^/ *R*_free_^d^ \| 0.1809/0.2269 \| \| No. atoms: Protein \| 2965 \| \| No. atoms: Water \| 349 \| \| No. atoms: Ligand \| 40 \| \| Wilson B \| 23.90 \| \| B factor (Å^2^) – All \| 29.01 \| \| B factor (Å^2^) – Water \| 34.42 \| \| B factor (Å^2^) – Protein \| 28.07 \| \| B factor (Å^2^) – Ligand \| 65.29 \| \| R.m.s. deviations \|  \| \| Bond lengths (Å) \| 0.007 \| \| Bond angles (°) \| 0.83 \| \| Ramachandran plot \|  \| \| Residues in most favored/additionally allowed regions (%) \| 97.85/2.15 \| \| MolProbity Score (percentile) \| 1.3 (99^th^) \|     **Supplementary Table 3**   \| Residue \| Phenylthiazole **1** atoms \| NMR^[a]^ \| Crystal^[c]^ \| \| --- \| --- \| --- \| --- \| \| Leu 40-Q^δ1^ \| H71/H72 \| 4.2 \| 4.5/5.6 \| \| Leu 40-Q^δ1^ \| H91/H92 \| 4.7 \| 5.3/6.1 \| \| Ile 42-Q^δ1^ \| H71/H72 \| 4.5 \| 8.1/9.4 \| \| Ile 42-Q^δ1^ \| H91/H92 \| 5.2 \| 7.6/9.0 \| \| Ile 42-Q^δ1^ \| H1/H5 \| 5.0 \| 8.5/9.3 \| \| M153-Q^ε^ \| H15/H19 \| 7.6 \| 14.3/15.7 \| \| T168-Q^γ2^ \| H71/H72 \| 4.9 \| 6.2/6.3 \| \| T168-Q^γ2^ \| H91/H92 \| 4.5 \| 3.5/4.0 \| \| T168-Q^γ2^ \| H15/H19 \| ND^[b]^ \| 4.4/7.2 \| \| T168-Q^γ2^ \| H16/H18 \| ND^[b]^ \| 6.3/8.5 \| \| M171-Q^ε^ \| H71/H72 \| 5.0 \| 6.7/7.7 \| \| M171-Q^ε^ \| H91/H92 \| 5.3 \| 9.5/9.5 \| \| M171-Q^ε^ \| H1/H5 \| 5.3 \| 4.6/8.2 \| \| M171-Q^ε^ \| H2/H4 \| 5.8 \| 3.7/7.7 \|   **Supplementary Table 4**   \|  \| HADDOCK_1 \| HADDOCK_2 \| HADDOCK_3 \| HADDOCK_4 \| HADDOCK_5 \| X-ray \| CYANA \| *N*MR^2^_2 \| *N*MR^2^_1 \| \| --- \| --- \| --- \| --- \| --- \| --- \| --- \| --- \| --- \| --- \| \| HADDOCK_1 \|  \| 2.523 \| 2.069 \| 1.66 \| 2.882 \| 4.263 \| 3.571 \| 1.181 \| 3.959 \| \| HADDOCK_2 \| 2.523 \|  \| 2.067 \| 2.119 \| 1.819 \| 4.598 \| 3.115 \| 2.71 \| 3.378 \| \| HADDOCK_3 \| 2.069 \| 2.067 \|  \| 2.37 \| 2.66 \| 3.752 \| 3.376 \| 2.646 \| 3.737 \| \| HADDOCK_4 \| 1.66 \| 2.119 \| 2.37 \|  \| 2.325 \| 4.077 \| 3.242 \| 1.755 \| 3.7 \| \| HADDOCK_5 \| 2.882 \| 1.819 \| 2.66 \| 2.325 \|  \| 4.801 \| 2.888 \| 2.791 \| 3.086 \| \| X-ray \| 4.263 \| 4.598 \| 3.752 \| 4.077 \| 4.801 \|  \| 3.313 \| 4.594 \| 3.725 \| \| CYANA \| 3.571 \| 3.115 \| 3.376 \| 3.242 \| 2.888 \| 3.313 \|  \| 3.561 \| 1.389 \| \| *N*MR^2^_2 \| 1.181 \| 2.71 \| 2.646 \| 1.755 \| 2.791 \| 4.594 \| 3.561 \|  \| 3.97 \| \| *N*MR^2^_1 \| 3.959 \| 3.378 \| 3.737 \| 3.7 \| 3.086 \| 3.725 \| 1.389 \| 3.97 \|  \|   Supplementary Fig. 1  **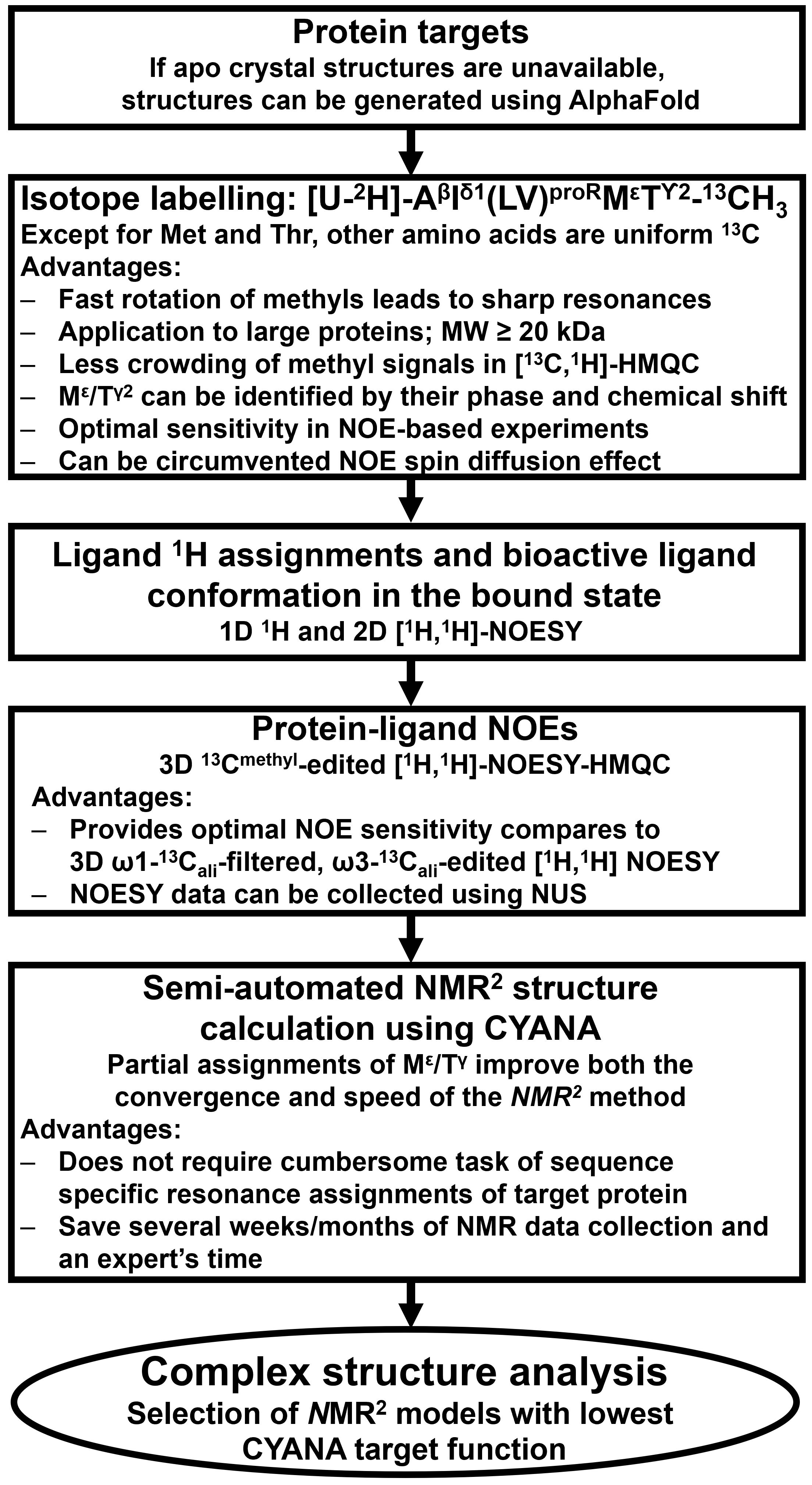** |
| --- | --- | --- | --- | --- | --- | --- | --- | --- | --- | --- | --- | --- | --- | --- | --- | --- | --- | --- | --- | --- | --- | --- | --- | --- | --- | --- | --- | --- | --- | --- | --- | --- | --- | --- | --- | --- | --- | --- | --- | --- | --- | --- | --- | --- | --- | --- | --- | --- | --- | --- | --- | --- | --- | --- | --- | --- | --- | --- | --- | --- | --- | --- | --- | --- | --- | --- | --- | --- | --- | --- | --- | --- | --- | --- | --- | --- | --- | --- | --- | --- | --- | --- | --- | --- | --- | --- | --- | --- | --- | --- | --- | --- | --- | --- | --- | --- | --- | --- | --- | --- | --- | --- | --- | --- | --- | --- | --- | --- | --- | --- | --- | --- | --- | --- | --- | --- | --- | --- | --- | --- | --- | --- | --- | --- | --- | --- | --- | --- | --- | --- | --- | --- | --- | --- | --- | --- | --- | --- | --- | --- | --- | --- | --- | --- | --- | --- | --- | --- | --- | --- | --- | --- | --- | --- | --- | --- | --- | --- | --- | --- | --- | --- | --- | --- | --- | --- | --- | --- | --- | --- | --- | --- | --- | --- | --- | --- | --- | --- | --- | --- | --- | --- | --- | --- | --- | --- | --- | --- | --- | --- | --- | --- | --- | --- | --- | --- | --- | --- | --- | --- | --- | --- | --- | --- | --- | --- | --- | --- | --- | --- | --- | --- | --- | --- | --- | --- | --- | --- | --- | --- |

Supplementary Fig. 2


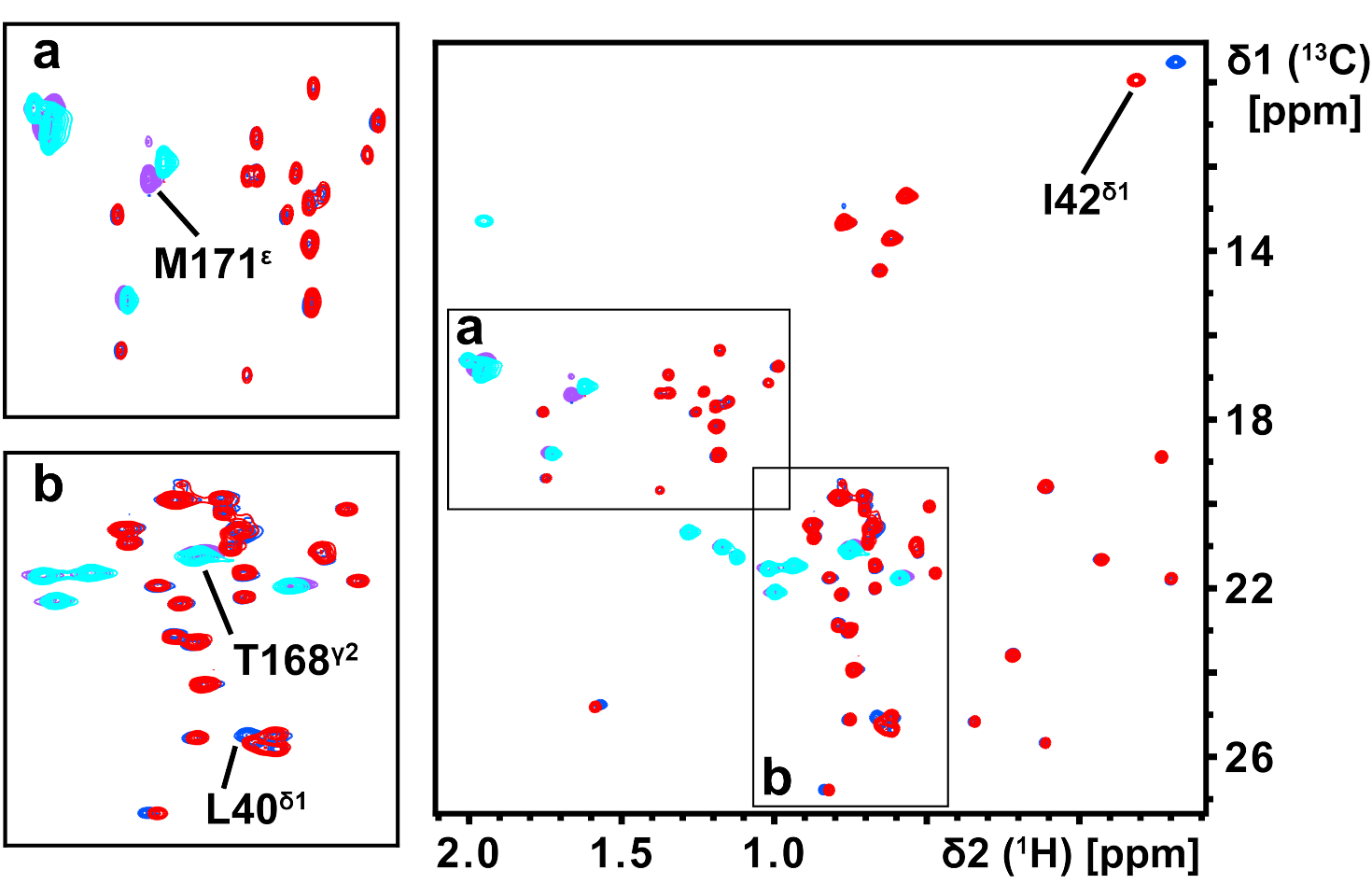


Supplementary Fig. 3

**
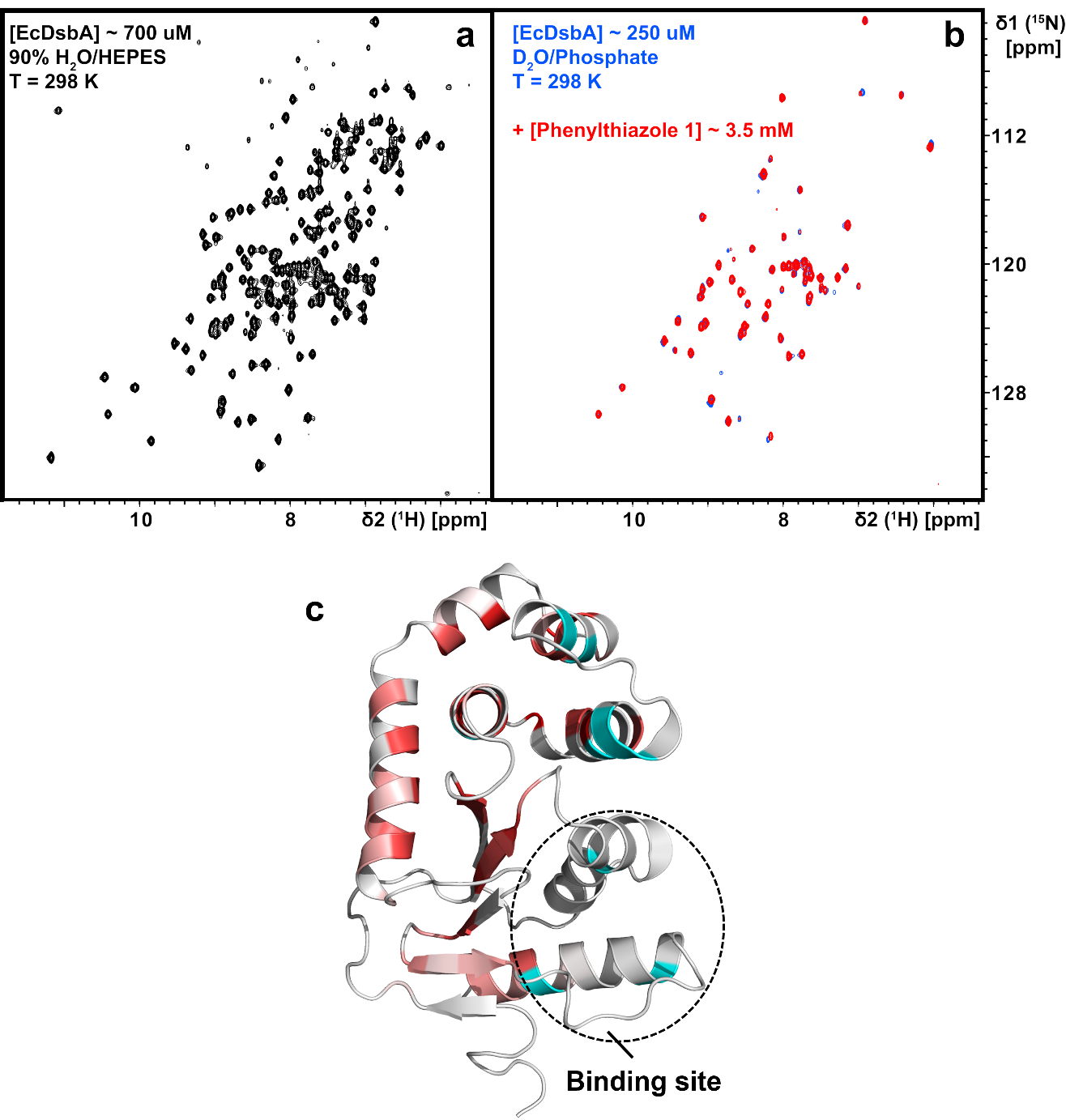
**

Supplementary Fig. 4


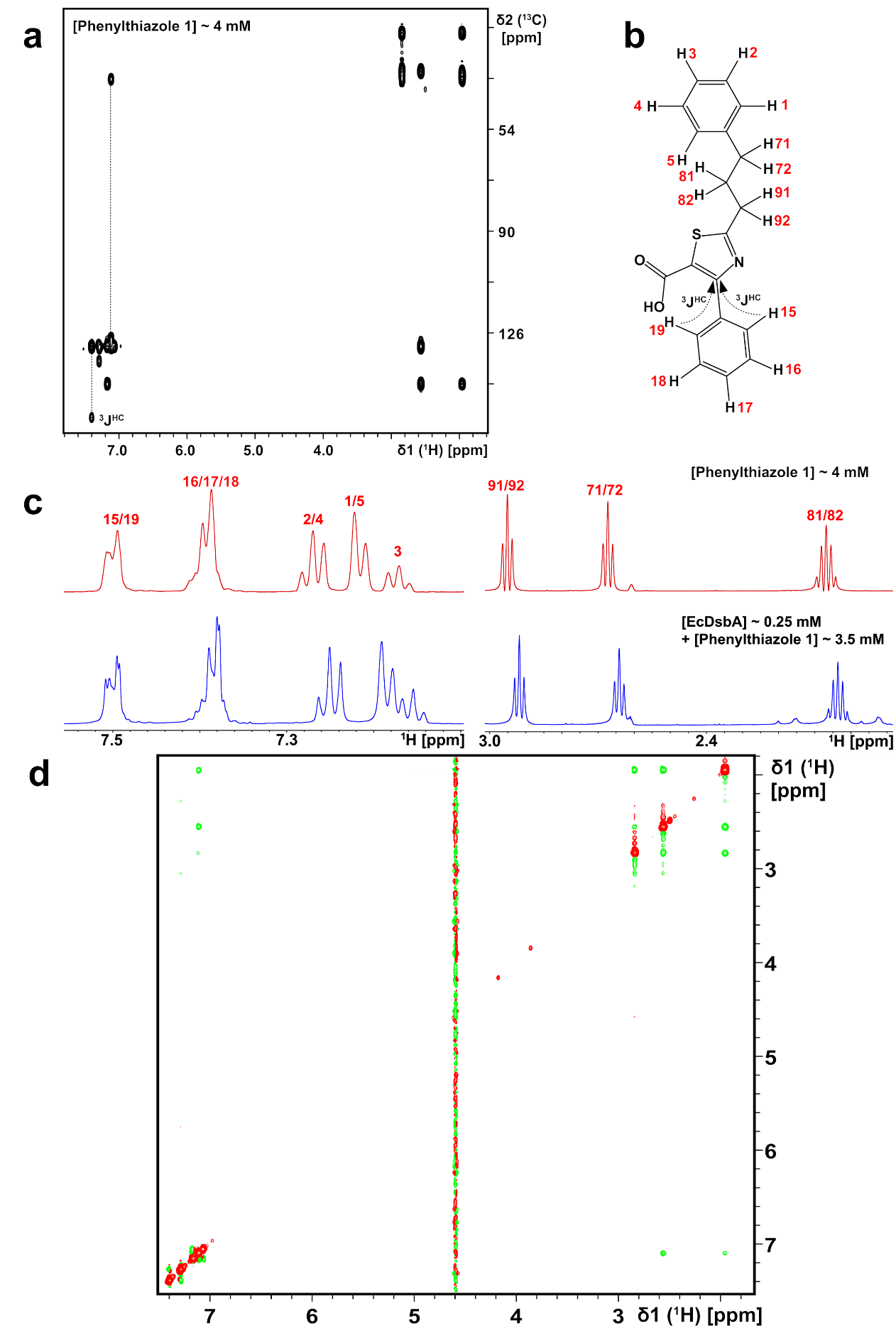


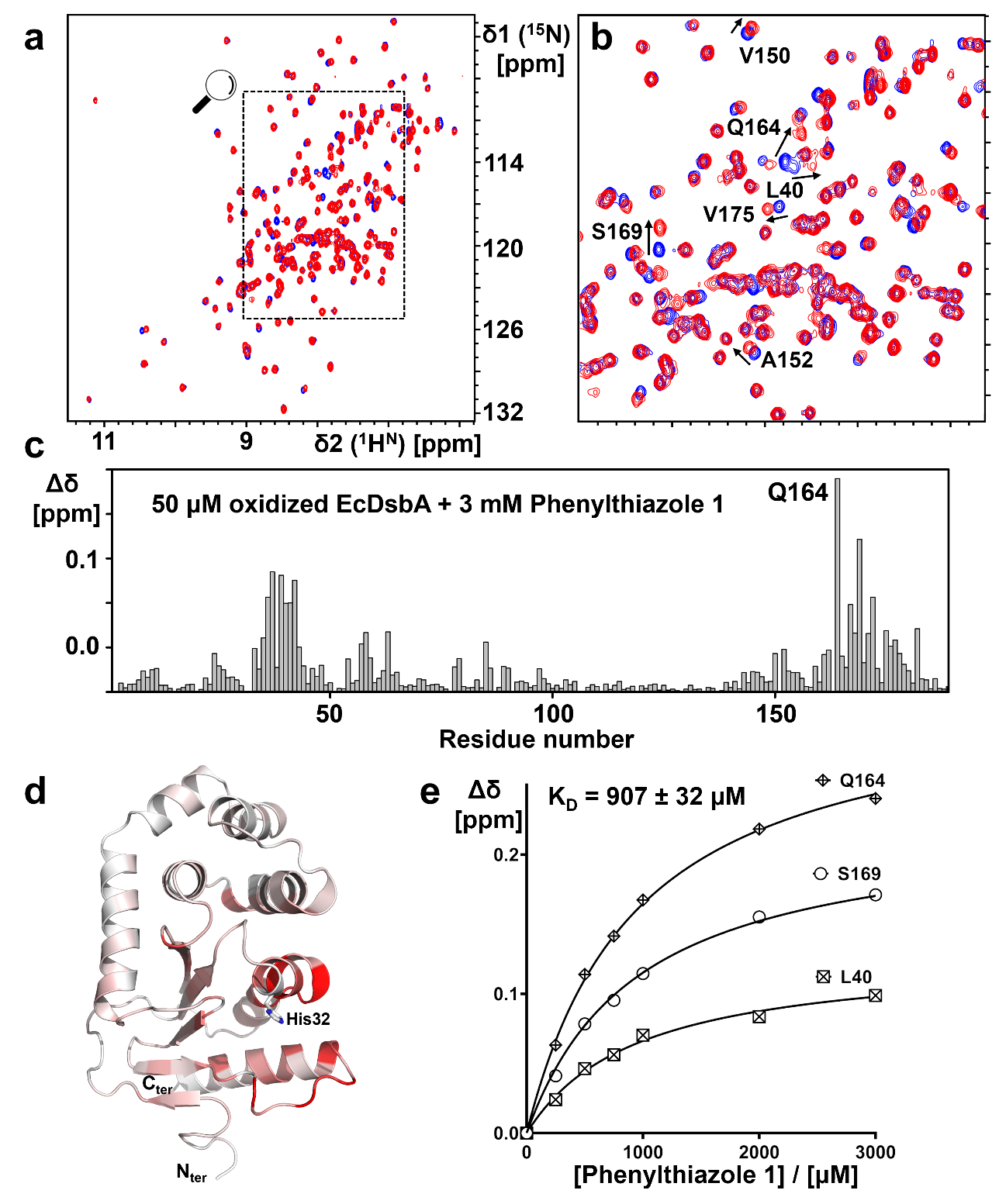
Supplementary Fig. 5

Supplementary Fig. 6


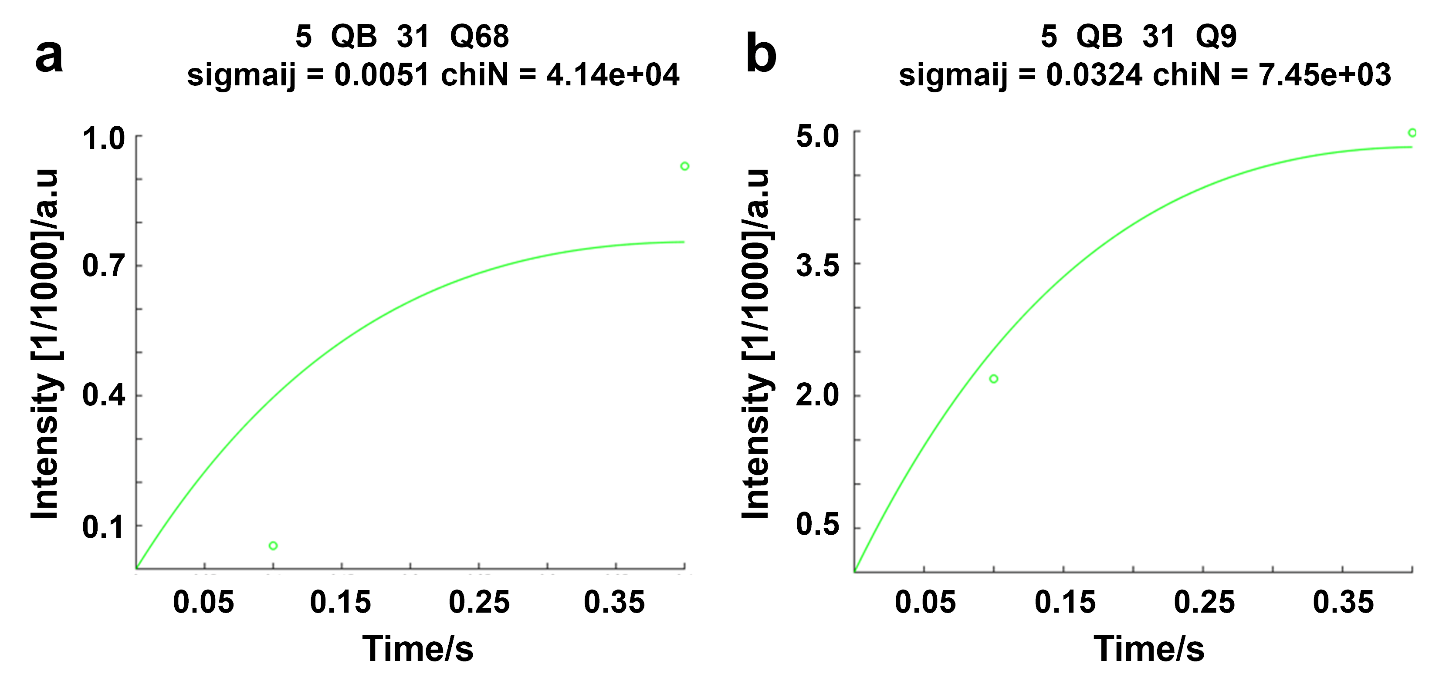


Supplementary Fig. 7

**
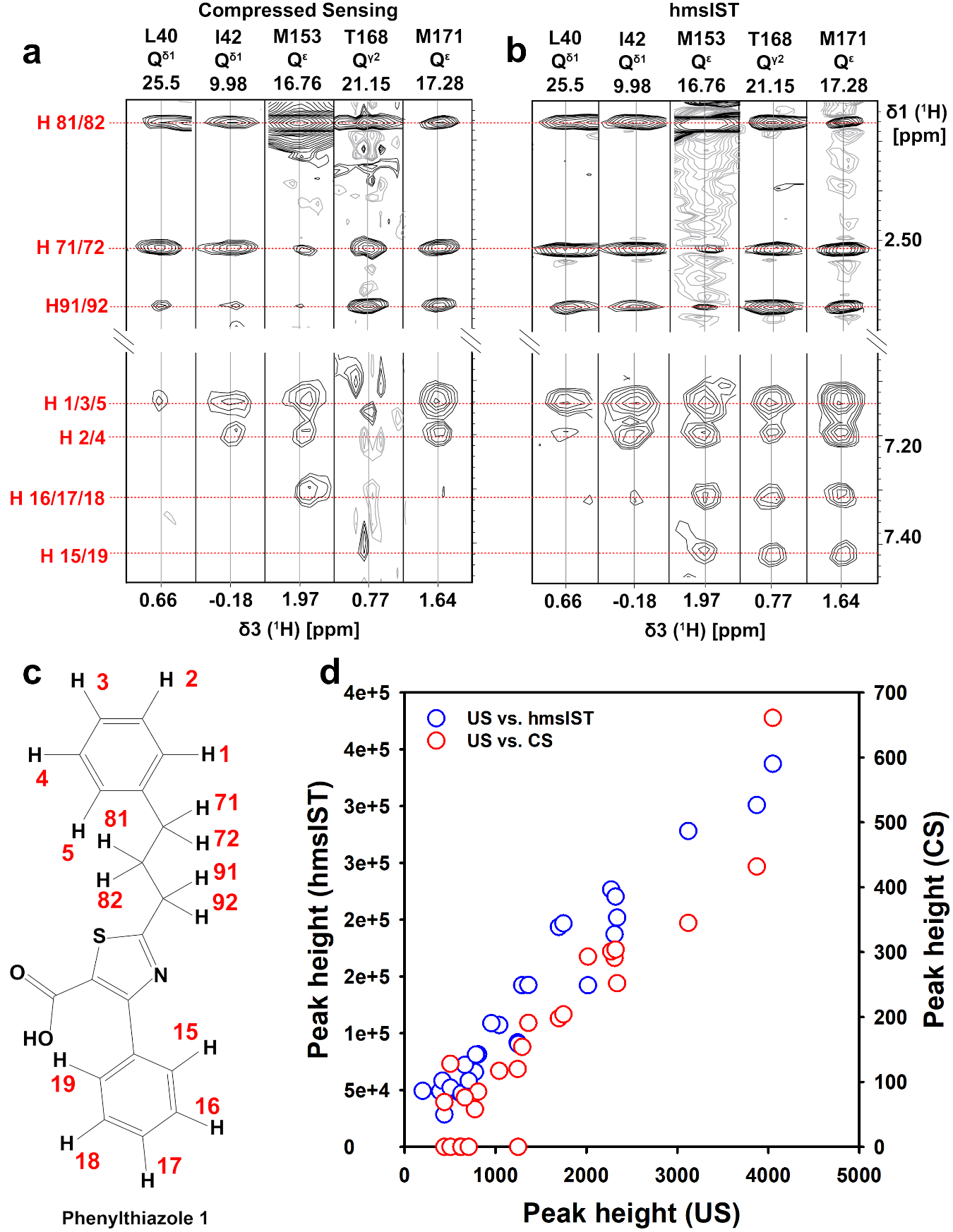
**

Supplementary Fig. 8


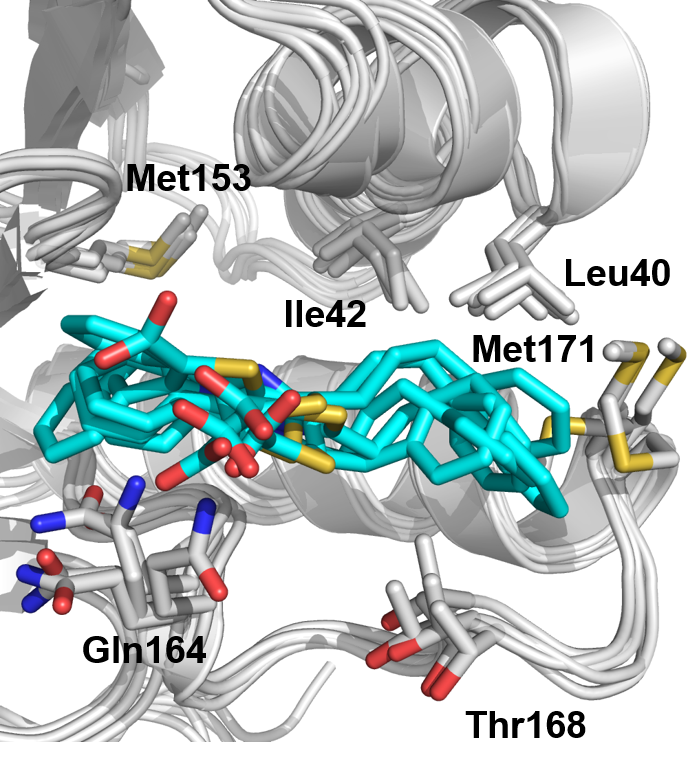

Supplement: Supplementary file 1 — Supplementary Information. [file 41598_2022_13561_MOESM1_ESM.docx]
